# Characterization of the Antimicrobial Activities of *Trichoplusia ni* Cecropin A as a High-Potency Therapeutic against Colistin-Resistant *Escherichia coli*

**DOI:** 10.3390/pharmaceutics15061752

**Published:** 2023-06-16

**Authors:** Hyeju Lee, Byeongkwon Kim, Minju Kim, Seoyeong Yoo, Jinkyeong Lee, Eunha Hwang, Yangmee Kim

**Affiliations:** 1 Department of Bioscience and Biotechnology, Konkuk University, Seoul 05029, Republic of Korea; hju0814@konkuk.ac.kr (H.L.); matt97@konkuk.ac.kr (B.K.); alswn7074@konkuk.ac.kr (M.K.); leejk809@konkuk.ac.kr (J.L.); 2 Center for Research Equipment, Korea Basic Science Institute, Cheongju 28119, Republic of Korea; hwang0131@kbsi.re.kr

**Keywords:** antimicrobial peptide, colistin-resistant *Escherichia coli*, lipopolysaccharide, *Trichoplusia ni* cecropin

## Abstract

The spread of colistin-resistant bacteria is a serious threat to public health. As an alternative to traditional antibiotics, antimicrobial peptides (AMPs) show promise against multidrug resistance. In this study, we investigated the activity of the insect AMP *Tricoplusia ni* cecropin A (*T. ni* cecropin) against colistin-resistant bacteria. *T. ni* cecropin exhibited significant antibacterial and antibiofilm activities against colistin-resistant *Escherichia coli* (ColREC) with low cytotoxicity against mammalian cells in vitro. Results of permeabilization of the ColREC outer membrane as monitored through 1-N-phenylnaphthylamine uptake, scanning electron microscopy, lipopolysaccharide (LPS) neutralization, and LPS-binding interaction revealed that *T. ni* cecropin manifested antibacterial activity by targeting the outer membrane of *E. coli* with strong interaction with LPS. *T. ni* cecropin specifically targeted toll-like receptor 4 (TLR4) and showed anti-inflammatory activities with a significant reduction of inflammatory cytokines in macrophages stimulated with either LPS or ColREC via blockade of TLR4-mediated inflammatory signaling. Moreover, *T. ni* cecropin exhibited anti-septic effects in an LPS-induced endotoxemia mouse model, confirming its LPS-neutralizing activity, immunosuppressive effect, and recovery of organ damage in vivo. These findings demonstrate that *T. ni* cecropin exerts strong antimicrobial activities against ColREC and could serve as a foundation for the development of AMP therapeutics.

## 1. Introduction

Gram-negative bacterial infections constitute a serious public health concern because they can cause extensive infections such as pneumonia, urinary tract infections, and bloodstream infections [1]. *Escherichia coli* (*E. coli*), a major cause of bacterial nosocomial infections, is treated with a variety of antibiotics and has acquired resistance to them. The production of extended-spectrum β-lactamase (ESBL) is a prominent resistance that interferes with the treatment of infections caused by *E. coli*. ESBL-producing *E. coli* acquires antibiotic resistance by hydrolyzing β-lactam antibiotics. There are various families of ESBLs, including temoniera (TEM), sulphydryl variable (SHV), and cefotaximase-Munich (CTX-M) types [2]. Carbapenems were chosen to treat ESBL-producing *E. coli*, which also developed resistance to carbapenems by producing carbapenemase [3]. *E. coli* have adapted to antibiotic environmental pressure, rendering them difficult to treat and often requiring the use of antibiotics of last resort such as colistin (polymyxin E), thereby posing a major global threat [4]. Polymyxins are polycationic antibiotics, first discovered in the 1940s, that exhibit potent activity against Gram-negative bacteria, including many that were resistant to other antibiotics [5]. However, despite their potent antibacterial activity, the use of polymyxins has been limited by their potential toxicity, including nephrotoxicity, neurotoxicity, and other adverse effects [6]. Owing to these concerns, the use of polymyxins declined in the 1980s, but with the emergence of multidrug-resistant bacteria, colistin was reconsidered as a treatment option [7].

Polymyxin B and colistin, the two types of polymyxins used in clinical practice, are polypeptides consisting of a heptapeptide ring connected to an N-terminal acylated tripeptide. The amino acids comprising the ring include D-Phe for polymyxin B, whereas colistin includes D-Leu in position 6 [8]. The mechanism of action of polymyxins involves binding to the negatively charged lipopolysaccharides (LPS) that are a component of the outer membrane of Gram-negative bacteria. LPS is composed of three domains: O-antigen, core oligosaccharide, and lipid A [9]. Polymyxins interact with LPS by binding to its lipid A portion, which is responsible for the endotoxic activity of LPS [10]. The hydrophilic part of polymyxins, which includes the cationic L-2,4-diaminobutyric acid (Dab), interacts with the negatively charged phosphate groups in the lipid A part of LPS [11]. This electrostatic interaction initially temporarily stabilizes the complex, allowing the N-terminal fatty acyl chain of the polymyxins to approach the outer membrane [12]. The hydrophobic tail of polymyxin inserts into the lipid A fatty acyl chain, eventually disrupting the integrity of the bacterial membrane. Hydrogen bonding interactions between the hydrophilic amino acids in polymyxins and the sugar moieties in LPS also contribute to the stability of the colistin–LPS complex [13]. Furthermore, the divalent cations (Ca^2+^, Mg^2+^) that provide additional stability to the outer membrane are substituted by colistin, leading to displacement of the cations by electrostatic interaction and resulting in disorganization of the bacterial membrane through the release of LPS [14]. Binding to LPS is essential for the antibacterial activity of polymyxins, which disrupts the integrity of the outer membrane of Gram-negative bacteria and ultimately leads to cell death [15].

Colistin is widely used clinically for the treatment of patients with Gram-negative bacterial infections; however, the emergence of colistin-resistant bacteria constitutes a growing threat, exacerbated by the lack of significant advances in the development of alternatives against bacterial infection. One of the main mechanisms by which Gram-negative bacteria develop colistin resistance is by modifying the composition of their outer membrane, mediated by bacterial adaptation to exposure to this antibiotic. This involves the alteration of LPS via cationic modification by adding a positively charged phosphoethanolamine moiety together with 4-amino-4-deoxy-L-arabinose to the 4′- and/or 1-phosphate of the lipid A part, resulting in a reduction in the electronegativity of the cell membrane. Further modifications of lipid A also include its acylation or deacylation [16]. In turn, the binding affinity of polymyxins toward LPS in colistin-resistant bacteria significantly decreases, resulting in the loss of their bactericidal activity [17,18]. Additionally, some bacteria can overexpress efflux pump systems that excrete colistin [19]. These mechanisms of resistance can emerge through spontaneous genetic mutations or be acquired through the transfer of resistance genes between bacteria [20]. Numerous studies are currently being conducted with the aim of restoring the activity of polymyxins against resistant strains by designing polymyxin derivatives. FADDI series derivatives, in which hydrophobicity was modified through the replacement of amino acids at residues 6 and 7 or by the substitution of an acyl chain, were developed by researchers at Monash University. These derivatives have been shown to exhibit improved binding to lipid A, resulting in increased activity relative to those of polymyxin B and colistin in polymyxin-resistant strains [21,22]. Ongoing clinical trials for various derivatives of polymyxin B, including SPR 206 (NCT number: NCT04868292), QPX9003 (NCT number: NCT04808414), and MRX-8 (NCT number: NCT04649541), are focused on reducing nephrotoxicity and improving pharmacokinetic properties to enhance clinical efficacy [23,24,25]. As such, developing alternative agents to counter colistin resistance constitutes an essential public health challenge.

Antimicrobial peptides (AMPs), which comprise innate immune molecules that combat microbials in various organisms, have come under the spotlight as an alternative potential tool to overcome the problem of antibiotic resistance [26]. AMPs typically function by disrupting bacterial membranes, leading to cell lysis and death [27]. This mechanism differs from that of conventional antibiotics, which typically target specific intracellular targets such as bacterial enzymes or proteins; AMPs also have immunomodulatory effects [28,29]. Insects in particular produce AMPs to strengthen their innate immune systems, countering their constant exposure to pathogens as a result of living in the wild; this results in high resistance to microbial infections. The first insect defense antimicrobial peptide, cecropin A (KWKLFKKIEKVGQNIRDGIIKAGPAVAVVGQATQIAK-NH 2), was isolated from the hemolymph of the moth *Hyalophora cecropia* and is classified as an α-helical AMP [30]. Subsequently, various cecropins have been isolated from insects such as the silk moth *Bombyx mori*, wax moth *Galleria mellonella*, Chinese oak silkmoth *Antheraea pernyi,* and Asian swallowtail *Papilio xuthus* [31,32,33,34]. Insect cecropins display potent antibacterial, antifungal, anticancer, and anti-septic activities [35,36,37,38,39,40,41]. We have determined the structure of an insect cecropin, papiliocin, containing a cationic amphipathic α-helix at the N-terminus and hydrophobic C-terminal helix connected by a hinge region, which are critical for bacterial membrane permeabilization as well as LPS interactions [42,43]. These structural components are generally conserved in insect cecropins [42,44,45].

The structural component composition involving separate amphipathic and hydrophobic parts of insect cecropins is similar to that of colistin. Consistent with this, we found that insect cecropins show antibacterial activity against Gram-negative bacteria and binding affinity to LPS comparable to those of polymyxin B and colistin; moreover, they also exhibit antibacterial activity against multidrug-resistant Gram-negative bacteria [37,46,47]. Here we further hypothesize that unlike for polymyxins, whose LPS-binding affinity is highly sensitive to the modified outer membrane LPS lipids in colistin-resistant Gram-negative bacteria, the antimicrobial activities of insect cecropins might not be disrupted by such LPS modifications as a consequence of their own evolutionary adaptation. Therefore, cecropins may represent potent candidates as new types of antibiotics against colistin-resistant Gram-negative bacteria.

In particular, the expression of *Trichoplusia ni* cecropin A (*T. ni* cecropin), a 38-mer AMP (RWKFFKKIEKVGQNIRDGIIKAGPAVAVVGQAASITGK-NH2) first isolated and sequenced by Kang et al. through differential display [48], is increased in *T. ni* larvae upon bacterial infection [49], suggesting its potential antimicrobial functionality. Notably, *T. ni* cecropin is unique among all known insect cecropins as it contains an additional Phe at the N-terminus, which might be important for its antibacterial activity. However, compared with those of other cecropins, the potential benefits of *T. ni* cecropin as an antimicrobial peptide remain to be elucidated. Because *colistin resistance* is most frequently observed in *E. coli* among various Gram-negative bacteria [50], in this study we investigated the antibacterial and anti-inflammatory activities of *T. ni* cecropin against colistin-resistant *E. coli* (ColREC). Furthermore, we elucidated the underlying antimicrobial mechanism of action through the analysis of LPS-neutralizing activities together with the evaluation of anti-septic activities in an in vivo mouse endotoxemia model. Together, our findings highlight the potential of *T. ni* cecropin in the fight against Gram-negative antibiotic-resistant bacteria.

## 2. Materials and Methods

### 2.1. Peptide Synthesis

All peptides (*T. ni* cecropin, cecropin A from *H. cecropia*, polymyxin B, colistin, LL-37, and melittin) were solid-phase synthesized using fluorenylmethoxycarbonyl (Fmoc) chemistry at Anygen Co., Ltd. (Gwangju, Republic of Korea). They were purified to >95% purity using high-performance liquid chromatography using a C18 column and characterized using matrix-assisted laser-desorption ionization-time-of-flight mass spectrometry (Figure S1).

### 2.2. Bacteria Strains

*E. coli* (KCTC 1682) was obtained from the Korean Collection for Type Cultures (KCTC, Jeongeup, Republic of Korea), and *Acinetobacter baumannii* (KCCM 40203) and *Pseudomonas aeruginosa* (KCCM 11328) from the Korea Culture Center of Microorganisms (KCCM, Seoul, Republic of Korea). *Klebsiella pneumoniae* (NCCP 16054), colistin-resistant *E. coli* NMA 1557 (ColREC 1557), NMS 12 (ColREC 12), and colistin-resistant *A. baumannii* NMS 1915 (ColRAB 1915), as well as colistin-resistant *K. pneumoniae* NMS 139 (ColRKP 139), were obtained from the National Institute of Health Multidrug Resistant Bacteria Specialized Pathogen Resources Bank (Osong, Republic of Korea).

### 2.3. Minimum Inhibitory Concentration (MIC)

The antibacterial abilities of the peptides (*T. ni* cecropin, cecropin A, polymyxin B, colistin, and melittin) were determined using the broth dilution method as previously reported [51]. The peptides dissolved at 10 mg/mL in deionized water were serially diluted from 64 μM to 0.5 μM in 96-well plates using Mueller–Hinton (MH) broth, and bacteria cultured to exponential growth were added at 2 × 10^5^ cells/mL. The group of bacteria that grew untreated in the broth was set as the positive control, and the uninoculated broth as the negative control. Dissolved peptides are colorless and equal to the OD value of the medium only. After 16 h incubation at 37 °C, the absorbance at 600 nm was measured using a SpectraMAX microplate reader (Molecular Devices, San Jose, CA, USA). Each optical density (OD) value was expressed as a percentage, with 100% growth for the positive control and 0% growth for the negative control, and the concentration that showed >95% killing was indicated as a MIC value.

### 2.4. Cytotoxicity

Murine macrophage RAW 264.7 cells and mouse fibroblast L-929 cells were used for research and were purchased from the Korea Cell Line Bank (Seoul, Republic of Korea). Both cells were cultured in Dulbecco’s Modified Eagle’s Medium (DMEM; Welgene, Gyeongsan, Republic of Korea) supplemented with 10% fetal bovine serum and 1% antibiotics (penicillin/streptomycin) at 37 °C in the presence of 5% CO_2_. To measure the cytotoxicities of peptides against RAW 264.7 cells and L-929 cells, both cell lines were seeded at a density of 1 × 10^5^ cells/well in a 96-well plate, to which each concentration of peptide solution (from 1.6 μM to 100 μM) was added, followed by incubation for 22 h. Subsequently, WST-8 (Biomax Co., Ltd., Guri, Republic of Korea) was added at 10% volume of the final solution, and the mixture was incubated for an additional 2 h. Absorbance was measured at 450 nm. Percentages were calculated based on untreated cells.

### 2.5. Hemolysis

Sheep red blood cells (sRBCs; KisanBio, Seoul, Republic of Korea) were added to three volumes of phosphate-buffered saline (PBS; 35 mM phosphate buffer containing 150 mM NaCl, pH 7.4) and centrifuged five times at 4 °C, 1000× *g* for 5 min, followed by a final suspension in PBS at 4% (*w*/*v*). Peptides serially diluted from 100 μM to 3.1 μM in PBS at 100 μL in a 96-well plate were prepared, and an equal volume of blood solution was added. After incubation at 37 °C for 1 h, the plates were centrifuged for 5 min at 4 °C, 1000× *g*. The absorbance of the supernatant was measured at 405 nm. The 100% hemolysis control was 0.1% Triton-X 100 added to the blood solution; the negative control was blood solution added to PBS.

### 2.6. Biofilm Inhibition Assay

To quantify the biofilm inhibitory activity of each peptide, ColREC 1557 and ColREC 12 were cultured in Luria–Bertani (LB) broth overnight and then sub-cultured in MH broth. Different concentrations of peptide (from 1 μM to 64 μM) were prepared in 96-well plates using MH broth (containing 0.2% glucose) and incubated with bacteria (2 × 10^5^ cells/mL) at 37 °C for 16 h. After incubation, the culture medium was removed and methanol was added as a fixative for 15 min. The completely dried plates were stained for 2 h by adding 100 μL of staining solution (0.1% (*w*/*v*) crystal violet in 0.25% (*v*/*v*) acetic acid). The residual staining solution was gently rinsed off three times using distilled water. Subsequently, 90% ethanol was used to dissolve the dye, and absorbance was measured at 600 nm. The untreated bacterial group was used as a control to compare biofilm production rates.

### 2.7. Bacteria Outer Membrane Permeability Test

The bacteria were grown to OD 0.6, washed three times with wash buffer (5 mM HEPES, 20 mM glucose, pH 7.4), and resuspended to OD 0.05 in the same buffer. To examine the permeability of the bacterial outer membrane by the peptide, 5 μM 1-N-phenylnaphthylamine (NPN) was added to 2 mL of bacteria suspension and monitored until the fluorescence intensity stabilized. This stabilized value was used as a control. Fluorescence was measured using a fluorescence spectrophotometer (Shimadzu Scientific Instruments, Kyoto, Japan) at excitation and emission wavelengths of 350 and 420 nm, respectively; the fluorescence intensity was checked by gradually increasing the peptide concentration (1, 2, 4, 6, and 8 μM). Fluorescence intensity data were analyzed after subtracting the fluorescence background level of NPN alone.

### 2.8. Circular Dichroism (CD) Analysis

To study the secondary structure of peptides, CD spectra of the peptides were collected using a J-810 spectropolarimeter (Jasco, Tokyo, Japan). Peptides (50 μM) in aqueous solution, 50 mM dodecylphosphorcholine (DPC), and 100 mm sodium dodecyl sulfate (SDS) micelles in a 1 mm path length cell were scanned three times with 0.1 nm intervals and recorded at 190 to 250 nm. Data were converted to mean residue ellipticity (θ) in deg∙cm^2^∙dmol^−1^ units as previously described [52].

### 2.9. Antimicrobial Activity Time-Course Assay

To determine the antibacterial activity of peptides over time, a 2 mL bacterial suspension was treated with *T. ni* cecropin at different concentrations (2 μM, 4 μM) and incubated at 37 °C. At each time point (5, 10, 15, 30, 45, 60, and 120 min), 100 μL of culture medium was smeared on LB agar plates; colonies were counted after 12 h of incubation at 37 °C.

### 2.10. Bacteria Morphology Imaging

Field emission-scanning electron microscopy (SU8020; Hitachi, Tokyo, Japan) was used to analyze changes in bacterial membrane morphology. Bacteria cultured to exponential growth were treated with peptides at 4 μM for 4 h, washed by PBS, fixed with 2.5% (*w*/*v*) glutaraldehyde overnight at 4 °C, further fixed with 1% osmium tetroxide, and dehydrated with progressively increasing concentrations of 50, 60, 70, 80, 90, and 100% ethanol solutions. The dehydration process was then extended by varying the volume ratio of ethanol to isoamyl acetate to 2:1, 1:1, and 1:2, followed by incubation in hexamethyldisilane for an additional 30 min. The dehydrated samples were then coated with platinum and imaged by scanning electron microscopy.

### 2.11. Limulus Amebocyte Lysate (LAL) Assay

To investigate the LPS-neutralizing activity, LAL assays were performed according to the protocol provided in the LAL assay kit (ToxinSensor™ Chromogenic LAL Endotoxin Assay Kit; GenScript, Piscataway, NJ, USA). Briefly, peptides diluted from 50 μM to 1.6 μM in LAL reagent water were incubated with LPS (2 ng/mL) for 10 min at 37 °C. LAL enzyme was then added and incubated under the same conditions. Substrate was added to react, after which three kinds of stop solutions were added in order. Absorbance (545 nm) was measured and quantified using an endotoxin standard graph.

### 2.12. BODIPY-TR-Cadaverine (BC) Displacement Assay

To measure the binding ability of peptides and LPS by concentration, fluorescent dye was prepared in 50 mM Tris buffer (pH 7.4) containing 50 μg/mL of LPS from *E. coli* O55:B5 (Sigma-Aldrich, St Louis, MO, USA) and 5 μg/mL of BC (ThermoFisher Scientific Inc., Waltham, MA, USA) and incubated for 6 h. Peptides were diluted from 50 μM to 1.6 μM using 50 mM tris buffer in a black 96-well plate, and an equal volume of fluorescent dye was added. After 30 min incubation, the activity was measured using a spectrofluorometer (Spectra Max Gemini; Molecular Devices) with an excitation wavelength of 580 nm and an emission wavelength of 620 nm.

### 2.13. Isothermal Titration Calorimetry (ITC)

To measure the binding affinity of a peptide to LPS, ITC experiments were performed using a MicroCal AutoiTC200 (Malvern Panalytical, Malvern, UK) at the Korea Basic Science Institute (KBSI, Ochang, Republic of Korea). *T. ni* cecropin (0.1 mM) was injected into 370 μL of 25 μM LPS (*E. coli* O111:B4, Sigma-Aldrich) in Dulbecco’s phosphate-buffered saline (DPBS, pH 7.0; Welgene) at 2.5 s intervals for 98 s at 37 °C for 38 injections. LPS was pretreated with 15 min vortex, 5 min heating at 60 °C, followed by 5 min sonication. The data were analyzed for binding affinity using MicroCal Origin software (MicroCal Origin, Northhampton, MA, USA).

### 2.14. Saturation Transfer Difference (STD)-Nuclear Magnetic Resonance (NMR)

To investigate the interaction between a peptide (0.5 mM) and LPS (O111:B4, 25 μM), STD-NMR spectra were obtained using a Bruker 700 MHz spectrometer (Bruker Biospin, Rheinstetten, Germany) at KBSI. Sample was dissolved in 20 mM sodium phosphate buffer (pH 5.9). Spectra were provided at −3.0 ppm with saturation of the LPS resonance selectively, and a reference spectrum at 40 ppm. STD-NMR data were acquired by subtracting off-resonances from the on-resonance spectrum with a cascade of 40 Gaussian-shaped pulses of 50 ms duration (total saturation time was 2 s) to obtain a difference spectrum. ^1^H chemical shifts of aromatic residues were assigned using NOESY (mixing time of 250, 350 ms) and TOCSY (mixing time of 70 ms) experiments.

### 2.15. Suppression of LPS-Induced Inflammatory Cytokines

Murine macrophage RAW 264.7 cells were used to measure the anti-inflammatory activities of peptides. Culture plates (96-well) were seeded at a density of 1 × 10^5^ cells/well and stimulated with 20 ng/mL LPS (O111:B4; Sigma-Aldrich) for 16 h after 1 h pretreatment with each concentration of peptide (0.6 to 10 μM). The control is an untreated cell. The culture supernatant was added to an equal volume of Griess reagent (Sigma-Aldrich), and the absorbance was measured at 540 nm. Nitrite production was quantified using a standard curve constructed using NaNO_2_.

Interleukin-6 (IL-6)-specific enzyme-linked immunosorbent assays (ELISA; R&D Systems, Minneapolis, MN, USA) were performed according to the method specified by the manufacturer. Briefly, cell culture supernatants were added to immune plates treated with the capture antibody, followed by the detection antibody and streptavidin-horseradish peroxidase. For each step, the plates were washed twice with PBS containing 0.05% Tween 20. The plates were incubated with tetramethylbenzidine substrate (Invitrogen, Carlsbad, CA, USA), and the reaction was stopped with 2 N H_2_SO_4_ to measure absorbance at 450 nm. A standard graph of each marker was plotted and quantified.

### 2.16. Suppression of Colistin-Resistant Bacteria-Induced Inflammatory Cytokines

To investigate the inflammatory response induced by ColREC 1557, RAW 264.7 cells (1 × 10^5^ cells/well) were treated with various concentrations of peptide (0.6 to 10 μM) 1 h before bacterial infection. The control was an untreated cell. ColREC 1557 that had been cultured in LB broth overnight and sub-cultured to the exponential phase were harvested, suspended in DMEM, and used to infect RAW 264.7 cells to a final concentration of 1 × 10^5^ cells/well. Subsequent nitrite and IL-6 detection was performed as described in Section 2.15.

### 2.17. Inhibition of Nitric Oxide (NO) Production by T. ni Cecropin in Response to Various Toll-like Receptors (TLRs)

RAW 264.7 cells, which express various TLRs, were used to investigate the inflammatory response to specific TLRs. Experiments were performed as previously described [53]. RAW 264.7 cells were seeded at 1 × 10^5^ cells/well in 96-well plates and stimulated with agonists of various TLRs after 1 h pretreatment with *T. ni* cecropin at each concentration. Agonists were purchased from Invivogen (San Diego, CA, USA), including Pam_2_CSK_4_ (TLR2/6), Pam_3_CSK_4_ (TLR1/2), LPS (O111:B4) (TLR4), imiquimod (TLR7), and ODN1826 (TLR9). NO production measurements were performed using a Griess reagent.

### 2.18. Secreted Embryonic Alkaline Phosphatase (SEAP) Assay

Human embryonic kidney (HEK)-Blue^TM^ hTLR4 (Invivogen) cells were prepared in HEK-Blue detection medium (Invivogen) to enable real-time detection of SEAP. The SEAP assay was performed as previously described [54]. Briefly, peptides were prepared by concentration in 96-well plates, and cells were added at 2.5 × 10^4^ cells/well. After 1 h, they were stimulated with LPS (O111:B4) (20 ng/mL), followed by 16 h incubation, and the 620 nm absorbance was measured.

### 2.19. Surface Plasmon Resonance (SPR)

Binding affinity measurements of the peptide to TLR4/myeloid differentiation factor 2 (MD-2) protein (R&D Systems) were performed on a Biacore T200 instrument (GE Healthcare, Danderyd, Sweden). The receptor was covalently bound to the carboxymethylated sensor chip surface using standard NHS/EDC coupling procedures. The sensor chip (Sensor Chip CM5; Cytiva, MA, USA) was loaded with 30 μg/mL of protein in sodium acetate buffer (pH 4.0) to a resonance value of 2500. Measurements were performed at a flow rate of 30 μL/min with increasing concentrations of peptide dissolved in PBS containing 0.05% Tween 20 at 25 °C. Analysis was performed using Biacore T200 Evaluation Software 3.0 (GE Healthcare, Chicago, IL, USA).

### 2.20. Flow Cytometry

Cell surface receptor and peptide interactions were investigated by flow cytometry. RAW 264.7 cells were pretreated with 10 μM *T. ni* cecropin, treated with LPS (O111:B4, 50 ng/mL) 30 min later, and incubated for 24 h. Harvested cells were blocked with 0.5% bovine serum albumin for 1 h. They were then incubated with anti-TLR4 antibody (ab13556, Abcam, Cambridge, MA, USA; 0.5 μg/1 × 10^6^ cells) for 20 min, followed by incubation with Alexa Fluor 546-conjugated secondary antibody (A-10040, Invitrogen; 1:200 dilution) for 20 min. Cold PBS washes were performed between each step. Cells were then suspended in 1% paraformaldehyde and analyzed using a CytoFlex flow cytometry analyzer (Beckman Coulter, Brea, CA, USA).

### 2.21. Animal Study Information

ICR mice (female, 6-week-old) were purchased from Orient Bio ( Seongnam, Republic of Korea). All mice were housed in specific pathogen-free conditions with controlled temperature and humidity. All procedures were approved by the Institutional Animal Care and Use Committee (IACUC) of Konkuk University, Seoul, Korea (IACUC number: KU22174).

### 2.22. Mouse Model of LPS-Induced Endotoxemia

ICR mice were randomly divided into four groups (three mice per group). Mock-treated “normal” animals were i.p. injected with PBS alone. The peptide control group was injected with *T. ni* cecropin (1 mg/kg); the LPS control group received LPS O127:B8 (18 mg/kg; Sigma-Aldrich). In the peptide treatment group, *T. ni* cecropin was injected 1 h prior to LPS injection. At 16 h post-injection, mice were euthanized, and serum was obtained for measurement of the levels of inflammatory cytokines IL-6 using ELISA kits (R&D Systems). The aspartate aminotransferase (AST), alanine aminotransferase (ALT), and blood urea nitrogen (BUN) levels in the serum were measured using standard kits from Asan Pharmaceutical (Seoul, Republic of Korea), as described previously [55].

### 2.23. Histological Analysis of Lung Tissue

Lungs were obtained after the euthanasia of mice in four groups, as described in Section 2.22. After obtaining tissue, it was washed twice in PBS, fixed in 4% (*v*/*v*) paraformaldehyde, and prepared as paraffin blocks for sectioning. After sectioning at a thickness of 6 mm, paraffin was deparaffinized with xylene. They were rehydrated with an ethanol concentration gradient and stained with hematoxylin and eosin. Lung sections were prepared on microscope slides and imaged with a light microscope (Eclipse Ni; Nikon, Tokyo, Japan).

### 2.24. Data Analysis

Data from experiments performed at least three times are presented as the mean ± standard error of the mean (SEM) of independent experiments. One-way and two-way analysis of variance (ANOVA) and Dunnett’s test were performed using GraphPad Prism software (GraphPad Software Inc., La Jolla, CA, USA). Values of *p* < 0.05 (*), *p* < 0.01 (**), *p* < 0.001 (***) were considered to represent statistically significant differences.

## 3. Results

### 3.1. Antibacterial Activities of T. ni Cecropin

The MIC of *T. ni* cecropin was examined to verify its antibacterial activities compared with those of cecropin A from *H. cecropia*, two polymyxins (polymyxin B and colistin), and melittin, a peptide that exhibits high antibacterial activity against Gram-negative bacteria [56]. Various standard Gram-negative bacteria (*E. coli*, *A. baumannii*, *P. aeruginosa*, and *K. pneumoniae*) and colistin-resistant bacteria (ColREC 1557, ColREC 12, ColRAB 1915, and ColRKP 139) were used for the measurement. MIC values are given in Table 1. *T. ni* cecropin possessed potent antibacterial activities. For all bacteria, *T. ni* cecropin showed superior antibacterial activities to those of melittin. Moreover, peptide activity was maintained regardless of antibiotic resistance. In addition, polymyxin B and colistin showed poor antibacterial activities against all ColREC, ColRAB, and ColRKP strains that acquired colistin resistance, whereas *T. ni* cecropin retained bactericidal activities against all colistin-resistant bacteria. Using the geometric mean (GM) value of the averaged MIC across all strains to assess bactericidal activity, *T. ni* cecropin had the best performance among the comparators with a mean MIC of 1.63, followed by cecropin A (2.25), melittin (14.25), polymyxin B (17.88), and colistin (33.72) (Table 1).

### 3.2. Toxicity of T. ni Cecropin to Mammalian Cells

**Figure 1:**
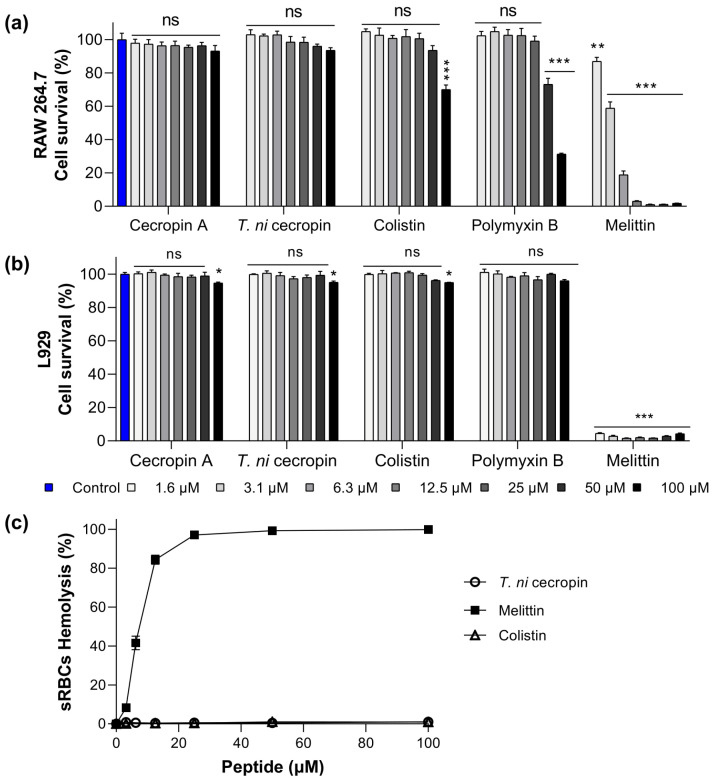
Cytotoxicity of peptides against (**a**) RAW 264.7 cells, (**b**) L929, and (**c**) sheep red blood cells (sRBCs). Data are presented as the mean ± SEM from triplicate experiments. * *p* < 0.05, ** *p* < 0.01, *** *p* < 0.001; and ns, nonsignificant compared to that in the non-treatment group (two-way analysis of variance).

To evaluate the potential use of *T. ni* cecropin as a drug, we obtained toxicity measurements in mammalian cells (murine macrophage RAW 264.7 cells and mouse fibroblast L-929 cells). In RAW 264.7 cells, melittin showed 3.1% cell viability at 12.5 μM (Figure 1a). Conversely, cells exposed to colistin and polymyxin B at a high concentration (100 μM) showed 70.1 and 31.3% viability, respectively, with *T. ni* cecropin exhibiting an outstanding survival rate of 93.6%. Similarly, in L-929 cells, the survival rate following melittin treatment was below 5% even at a concentration of 1.6 μM, whereas *T. ni* cecropin maintained 95% viability up to 100 μM (Figure 1b). Colistin (95.0%) and polymyxin B (96.0%) also showed similar survival rates. These results confirmed the safety of *T. ni* cecropin and indicated that melittin, which showed strong antimicrobial activity, is highly toxic to mammalian cells.

To further evaluate the toxicity of the peptides to mammalian cells, we investigated their hemolytic activity against sRBCs. Melittin showed substantial hemolytic activity (8.2%) at 3.1 μM and 84.3% at 12.5 μM, whereas *T. ni* cecropin and colistin showed no hemolytic activity even at 100 μM (Figure 1c). Together, the results of these toxicity studies confirmed the biocompatibility of *T. ni* cecropin with mammals. The relative selective index value was highest for *T. ni* cecropin (123.08), which was significantly higher than that for colistin (5.93) (Table 1).

### 3.3. T. ni Cecropin Inhibits ColREC Biofilm Formation

**Figure 2:**
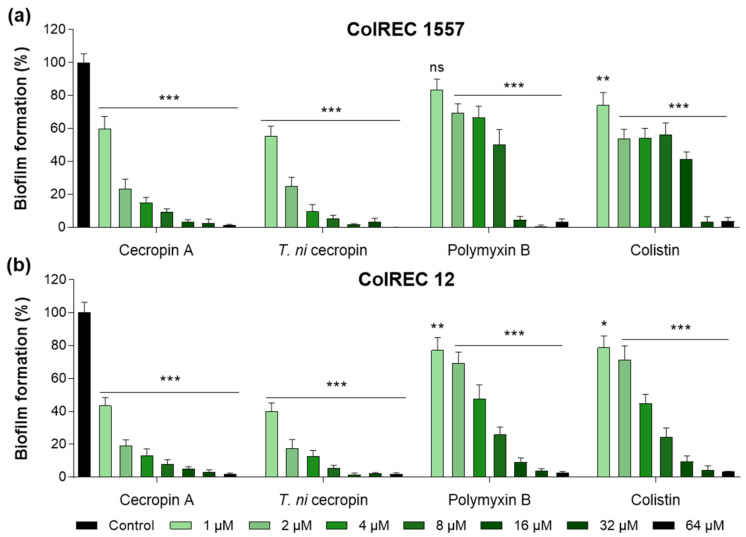
Inhibitory effects of peptides in biofilm assays performed in (**a**) ColREC 1557 and (**b**) ColREC 12. Data are presented as the mean ± SEM from triplicate experiments. * *p* < 0.05, ** *p* < 0.01, *** *p* < 0.001; and ns, nonsignificant compared to that in the non-treatment group (two-way ANOVA).

Biofilms create a growth environment for bacteria and are a cause of antibiotic resistance. To determine the effectiveness of the peptides against ColREC, biofilm formation inhibition was measured. The cecropins were superior to the polymyxins in antibiofilm activity. For ColREC 1557, *T. ni* cecropin showed 75.3% inhibition even at 2 μM and over 90% inhibition at 4 μM. In contrast, colistin prevented only 58.6% of the biofilm formation of ColREC 1557 at 16 μM (Figure 2a). Moreover, whereas 2 μM of colistin inhibited 28.9% of the biofilm formation in ColREC 12, the same concentration of *T. ni* cecropin and cecropin A had a higher inhibition rate of 82.5% and 80.8%, respectively (Figure 2b). This showed that *T. ni* cecropin as well as cecropin A interfere with the growth environment of colistin-resistant bacteria.

### 3.4. Antibacterial Mechanisms of T. ni Cecropin against Gram-Negative Bacteria

#### 3.4.1. LPS-Neutralizing Capacity of *T. ni* Cecropin

**Figure 3:**
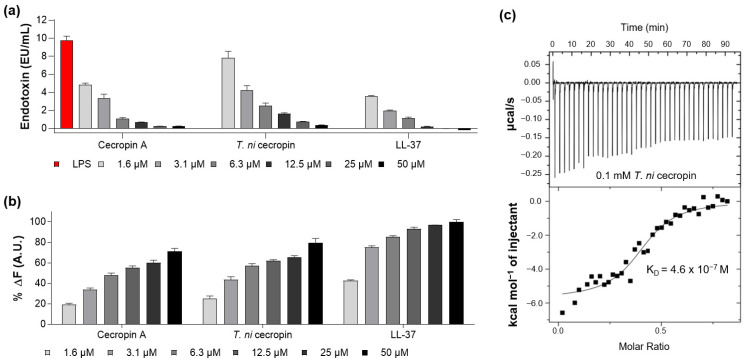
Measurement of interaction between lipopolysaccharide (LPS) and peptides. (**a**) Limulus amebocyte lysate assay showing the LPS neutralization capacities of peptides. (**b**) BODIPY-TR-cadaverine displacement from LPS after treatment with peptides. (**c**) Isothermal titration calorimetry measurement showing the binding affinity of *Trichoplusia ni* cecropin (*T. ni* cecropin) (0.1 mM) to 0.025 mM LPS. Data are presented as the mean ± SEM from triplicate experiments. The concentration of the peptides at each x-axis is indicated at the bottom of (**a**,**b**) by the color notation.

As the peptide forms a strong bond with LPS, it can suppress endotoxin toxicity caused by bacterial infection and lower the incidence of disease. The ability of the peptide to neutralize LPS was measured by quantifying the colorimetric reaction, indicating that LPS catalyzes proenzyme activation in LAL. LL-37, a strong LPS-neutralizing peptide, was used as a control [26]. The LAL test showed that *T. ni* cecropin and cecropin A reacted with LPS in a concentration-dependent manner, with 50 μM of the peptide nearly neutralizing 10 enzyme units (EU) of LPS (Figure 3a), as compared with the 12.5 μM required for LL-37.

The BC displacement assay was used to measure the ability of peptides to interact with LPS. BC fluorescent dyes quench when bound to cell-free LPS and emit fluorescence when LPS binds to the peptide. As shown in Figure 3b, *T. ni* cecropin induced concentration-dependent BC displacement. Based on a 100% displacement rate for 50 μM of LL-37, that for 50 μM of *T. ni* cecropin and cecropin A was 79.7% and 71.3%, respectively. These results demonstrate that both cecropins exhibit similar LPS neutralization and binding interactions as those of LL-37.

ITC analysis revealed that the binding affinity of *T. ni* cecropin to LPS was as high as 4.6 × 10^−7^ M, indicating an exothermic process with strong electrostatic interactions (Figure 3c). These results showed that *T. ni* cecropin directly interacts with LPS and neutralizes LPS, contributing to its antibacterial activity against Gram-negative bacteria.

#### 3.4.2. Membrane Depolarization Ability of *T. ni* Cecropin against *E. coli*

**Figure 4:**
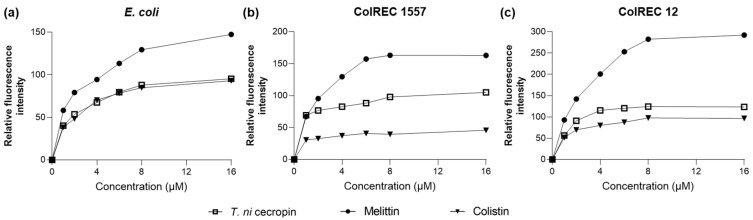
Fluorescence intensity as measured via peptide-induced membrane disruption. Relative fluorescence intensities in (**a**) *E. coli*, (**b**) ColREC 1557, and (**c**) ColREC 12 as measured using 1-N-phenylnaphthylamine uptake.

As we found that *T. ni* cecropin strongly interacted with LPS, we used the NPN uptake assay to measure the ability of the peptides to depolarize the outer membranes of *E. coli* and ColREC. NPN is a hydrophobic fluorescent probe that fluoresces in a hydrophobic environment and is used as an indicator of partitioning of the outer membrane. As shown in Figure 4, a dose-dependent increase in fluorescence intensity was observed upon treating each bacterium with various concentrations of peptide. For *E. coli*, colistin and *T. ni* cecropin showed similar permeation rates, whereas in resistant bacteria, *T. ni* cecropin permeated the membrane more efficiently than colistin. This provides evidence that *T. ni* cecropin can overcome resistance mechanisms by disrupting the integrity of the outer membrane of colistin-resistant bacteria.

#### 3.4.3. *T. ni* Cecropin Induces *E. coli* Cell Membrane Damage

**Figure 5:**
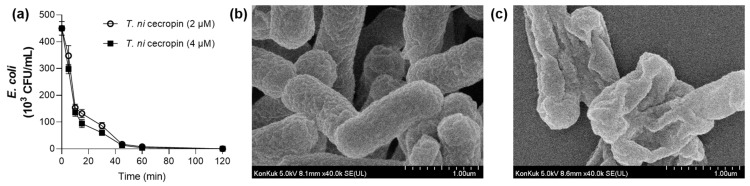
(**a**) Time-dependent killing activity of *T. ni* cecropin. Field emission-scanning electron microscopy images showing the morphology of *E. coli* treated with *T. ni* cecropin. (**b**) Untreated *E. coli* with intact morphology, and (**c**) after incubation for 4 h with *T. ni* cecropin at 4 μM (2 × MIC). Data are presented as the mean ± SEM from triplicate experiments.

The optimal duration of peptide exposure to *E. coli* was determined by considering the killing rate of the bacteria over time. For *T. ni* cecropin at 2 μM (1 × MIC) and 4 μM (2 × MIC), live bacteria were completely eradicated by 2 h incubation (Figure 5a). As shown in Figure 5b, exposure of *E. coli* to 4 μM of *T. ni* cecropin for 4 h caused substantial bacterial membrane damage. The control bacteria maintained a smooth and plump shape (Figure 5b) whereas those exposed to the peptide showed a distorted shape and obvious changes such as membrane contraction (Figure 5c). These data visually demonstrated that the peptides cause damage to the membrane and the presumed leakage of internal substances. Peptides may allow the efflux of internal cytoplasmic components through disruption of cytoplasmic membrane integrity, eventually leading to cell death. These results confirmed that *T. ni* cecropin shows antibacterial activity via a membrane disruption mechanism to resist microorganisms.

#### 3.4.4. *T. ni* Cecropin Directly Interacts with LPS

**Figure 6:**
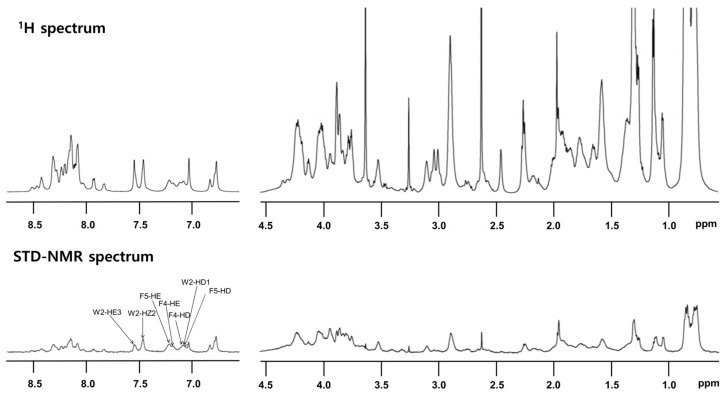
Reference one-dimensional nuclear magnetic resonance (NMR) spectrum for *T. ni* cecropin (**top**) and saturation transfer difference (STD) NMR spectrum obtained through interaction with LPS (**bottom**) dissolved in 20 mM sodium phosphate buffer (pH 5.9). Two regions corresponding to amide protons and aromatic ring protons (left side from 6.5 ppm to 9 ppm) and aliphatic protons (right side from 0.5 ppm to 4.5 ppm) are shown.

STD-NMR analysis was performed to specify the residues that interact with LPS. Peptide (0.5 mM) was reacted with 15 μM LPS. Notably, despite its high sequence homology with other cecropins, *T. ni* cecropin uniquely harbors an additional Phe at the fourth residue, substituting for Leu; thus, *T. ni* cecropin contains one Trp and two Phe residues. This difference may induce a strong interaction between *T. ni* cecropin and the bacterial membrane. The top spectrum in Figure 6 shows the reference spectrum of the free peptide, whereas the spectrum at the bottom shows the STD effect of the peptide bound to LPS (Figure 6 bottom). STD-NMR data showed that aromatic and amide protons, as well as aliphatic protons, contribute to the LPS interaction. Especially strong STD effects were identified at 7.0–7.7 ppm, corresponding to the aromatic ring protons of the Trp2, Phe4, and Phe5 shown in Figure 6, implying that these aromatic residues are important for interaction with LPS and the antibacterial activities of *T. ni* cecropin.

#### 3.4.5. Secondary Structure of *T. ni* Cecropin

**Figure 7:**
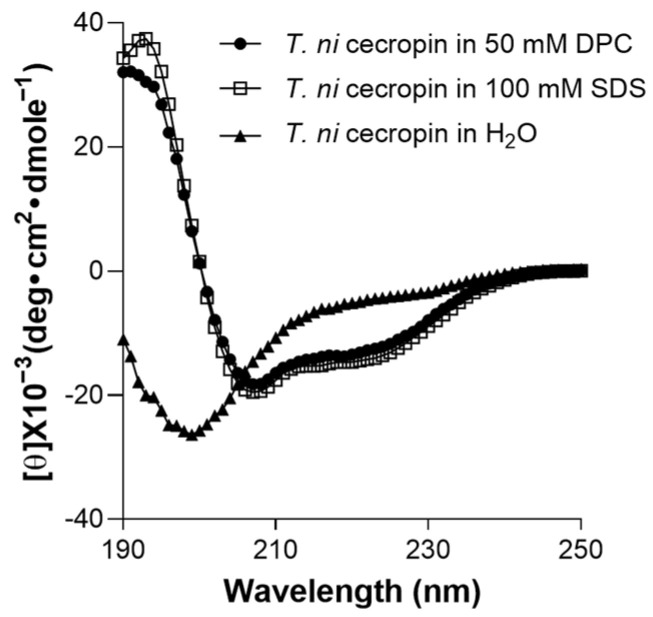
Secondary structures of *T. ni* cecropin in membrane-mimetic environments as observed via circular dichroism spectroscopy. DPC: dodecylphosphorcholine; SDS: sodium dodecyl sulfate.

To determine the environment-dependent structure of the peptide, CD spectrum analysis was performed. *T. ni* cecropin was not structured in aqueous solution (Figure 7). However, in SDS micelles, which mimic negatively charged bacterial cells, or in DPC micelles, which mimic zwitterionic cell membranes, it was observed to adopt a helical structure with strong positive values at 192 nm and double minima at 208 and 222 nm, equivalent to the characteristics of the α-helical structures of cecropin A [57].

### 3.5. Inhibition of Cytokine Production in RAW 264.7 Cells Stimulated by LPS or ColREC

**Figure 8:**
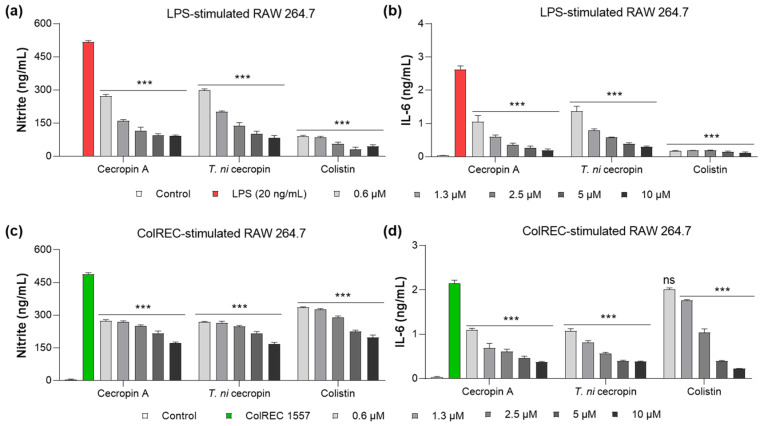
Anti-inflammatory effect in RAW 264.7 cells induced by stimulants. Graphs show the dose-dependent nitrite and interleukin-6 (IL-6) inhibitory effects of the peptide (**a**,**b**) on LPS stimulation and (**c**,**d**) ColREC stimulation. Data are presented as the mean ± SEM from triplicate experiments. *** *p* < 0.001 and ns, nonsignificant compared to that in the LPS or ColREC 1557 treatment group (two-way ANOVA).

LPS is a key virulence molecule of Gram-negative bacteria that stimulates an inflammatory cascade in macrophages. As part of the inflammatory response, stimulated cells secrete cytokines such as NO and IL-6 to regulate the inflammatory response. To evaluate whether *T. ni* cecropin, which we demonstrated to bind directly to LPS, has anti-inflammatory properties against LPS-induced inflammation, we measured its ability to inhibit cytokine production. The anti-inflammatory ability of colistin was evaluated in parallel. NO production was measured using the Griess assay to detect NO_2_^−^, whereas IL-6 was quantified using a sandwich ELISA. The untreated cells used as controls showed little cytokine release. NO was inhibited by 80.1% in LPS-stimulated RAW 264.7 cells upon the addition of 5 μM of *T. ni* cecropin (Figure 8a). At the same concentration, IL-6 secretion was reduced by 86.5% (Figure 8a,b). Cecropin A (5 μM) yielded similar outcomes as *T. ni* cecropin, with 81.2% and 91.0% inhibition of NO and IL-6, respectively. At the same concentration, colistin showed over 90% inhibition of both cytokines.

Moreover, *T. ni* cecropin showed superior activity with regard to antibacterial activity against ColREC, the direct cause of the infection, compared with that of colistin. Therefore, we aimed to identify differences in activity in the inflammatory response in macrophages infected with ColREC. RAW 264.7 cells were stimulated with ColREC, and the effect on cytokine production was measured using ELISA. Overall, cecropins showed enhanced anti-inflammatory activity compared with that of colistin, with predominant activity at lower concentrations (Figure 8c,d). NO and IL-6 were inhibited by 46.2 and 63.0%, respectively, upon the addition of 1.3 μM *T. ni* cecropin, whereas colistin showed much lower inhibition rates of 24.7 and 18.4% (Figure 8c,d). These results confirmed that *T. ni* cecropin can effectively counteract the inflammatory response induced by colistin-resistant bacteria even at concentrations as low as 0.6 μM.

### 3.6. T. ni Cecropin Selectively Targets the TLR4-Inflammatory Signaling Pathway

**Figure 9:**
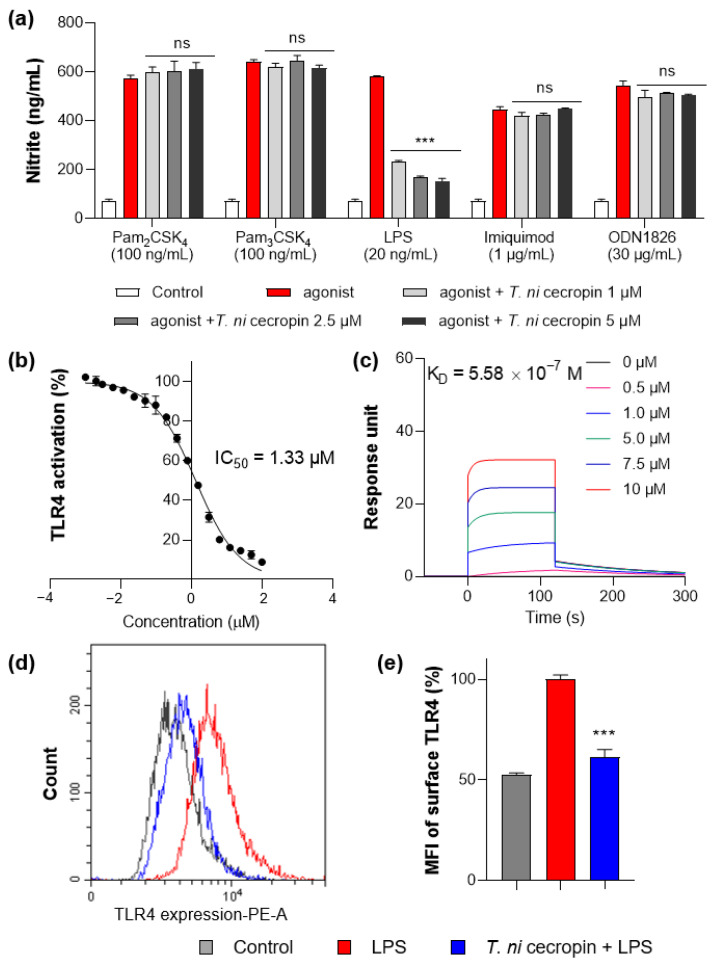
Measurement of interaction between Toll-like receptor 4 (TLR4) and peptides. (**a**) Specific agonist treatment for each TLR receptor shows TLR4 selectivity of *T. ni* cecropin. (**b**) Results of secreted embryonic alkaline phosphatase assay show the TLR4 inactivating effect of *T. ni* cecropin on LPS-stimulated human embryonic kidney-Blue hTLR4 cells. (**c**) Surface plasmon resonance sensorgrams of TLR4/MD-2 complex protein interaction with varying concentrations of *T. ni* cecropin. (**d**) Flow cytometry results showing the effect of *T. ni* cecropin on preventing TLR4 expression in LPS-stimulated cells and (**e**) indicating the mean fluorescence intensity (MFI) (%). Data are presented as the mean ± SEM from triplicate experiments. *** *p* < 0.001 and ns, nonsignificant compared to that in the agonist treatment group (**a**) (one-way ANOVA); *** *p* < 0.001 compared to that in the LPS group (**e**) (two-way ANOVA).

To understand the mechanism by which *T. ni* cecropin inhibits inflammation, we applied agonists for various TLRs to macrophage RAW 264.7 cells and evaluated their specificity against TLR proteins. Pam_3_CSK_4_, Pam_2_CSK_4_, LPS, imiquimod, and ODN 1826 were used as agonists for TLR2/1, TLR2/6, TLR4, TLR7, and TLR9, respectively. Only the group stimulated by LPS, an agonist for TLR4, showed cecropin-induced inhibition of inflammation (Figure 9a). *T. ni* cecropin had no effect on stimulation by other agonists, indicating that *T. ni* cecropin specifically targets TLR4.

To determine the specific action of *T. ni* cecropin on TLR4, we utilized HEK-Blue hTLR4 cells, which express the NF-κB-inducible SEAP reporter gene upon stimulation with LPS. Following 20 ng/mL LPS stimulation, *T. ni* cecropin treatment significantly reduced TLR4-mediated SEAP gene expression with a low half-maximal inhibitory concentration (IC_50_) of 1.33 μM (Figure 9b). This supports the model that *T. ni* cecropin specifically targets TLR4 signaling.

We next investigated the interaction between the TLR4/MD-2 complex and *T. ni* cecropin by performing SPR (Figure 9c). The protein complex was coated on a sensor chip and probed with various concentrations of peptide. *T. ni* cecropin bound to TLR4/MD2 in a concentration-dependent manner with high affinity, having an equilibrium constant (K_D_) of 5.58 × 10^−7^ M.

We next used flow cytometry to examine whether the expression level of the TLR4 receptor presented on the surface of RAW 264.7 macrophages was changed by *T. ni* cecropin (Figure 9d). The fluorescence of LPS-stimulated compared with LPS-untreated RAW 264.7 cells was measured using a fluorescent antibody attached to an anti-TLR4 antibody. Cells stimulated with LPS (red) expressed TLR4 on their surface and showed higher fluorescence compared to that of unstimulated control cells (gray). Cells co-treated with *T. ni* cecropin and LPS (blue) showed a significant decrease in TLR4 expression compared with that from LPS treatment alone, as confirmed by evaluating relative expression (61.4%) in LPS/*T. ni* cecropin-co-treated cells based on normalizing the fluorescence intensity from LPS-stimulated cells to 100% (*p* < 0.001). These results suggest that *T. ni* cecropin interferes with LPS binding to TLR4 and may blunt TLR4 surface presentation on macrophages.

### 3.7. T. ni Cecropin Significantly Attenuates LPS-Induced Endotoxemia in a Mouse Model

**Figure 10:**
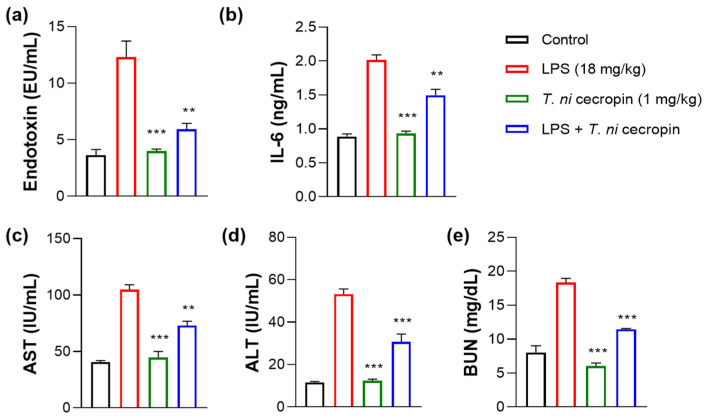

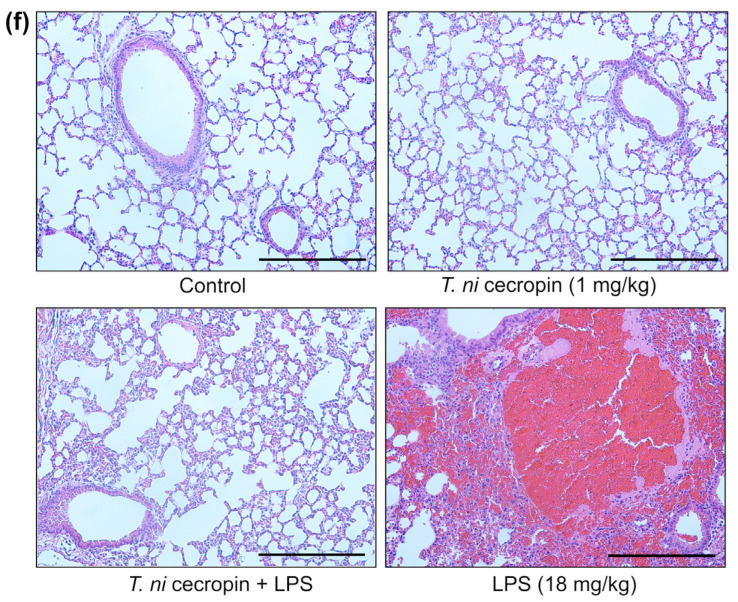
Anti-septic effects of *T. ni* cecropin in an LPS-induced endotoxemia septic shock mouse model. Suppressive effects on circulating serum (**a**) endotoxin levels and (**b**) IL-6 in the endotoxemia mouse model. (**c**–**e**) Recovery of aspartate aminotransferase (AST), alanine aminotransferase (ALT), and blood urea nitrogen (BUN) levels in the serum of the LPS-stimulated endotoxemia mouse model following treatment with *T. ni* cecropin. Data are presented as the mean ± SEM (n = 3 per group). ** *p* < 0.01 and *** *p* < 0.001 compared to that in the control group (one-way ANOVA). (**f**) Micrograph of lung histology for control, *T. ni* cecropin, LPS, and pre-treatment *T. ni* cecropin groups (clockwise) as indicated at the bottom of each photograph. Bars: 100 μm; 20× magnification.

We investigated the ability of *T. ni* cecropin to attenuate LPS-induced endotoxemia. *T. ni* cecropin treatment significantly reduced the endotoxin levels in an LPS-induced endotoxemia model, which agrees well with its LPS-neutralizing activities as shown via the in vitro LAL assay (Figure 3a and Figure 10a). Next, we tested the ability of *T. ni* cecropin to suppress pro-inflammatory cytokine production in the serum. Whereas LPS induced a marked increase in cytokines, pretreatment with *T. ni* cecropin downregulated LPS-induced cytokine levels by 46.1% for IL-6, which agrees well with its in vitro anti-inflammatory effects in LPS or ColREC-stimulated RAW 264.7 cells (Figure 8b,d and Figure 10b). Furthermore, we investigated the ability of *T. ni* cecropin to reduce LPS-induced organ damage as measured by serum AST, ALT, and BUN levels. An increase in AST and ALT levels induced by LPS detected in the serum indicates liver damage, while an elevation of BUN levels induced by LPS indicates kidney damage. *T. ni* cecropin elicited significant suppression of LPS-induced AST, ALT, and BUN levels, showing reductions of 49.5, 54.2, and 66.7%, respectively (Figure 10c–e). In the lungs, the administration effect of *T. ni* cecropin was observed by microscopy of tissue stained with hematoxylin and eosin. As shown in Figure 10f, alveolar structure destruction, hemorrhage, and neutrophilic infiltration were observed in LPS-injected mice. These damages were not observed in the other groups. The results suggest that *T. ni* cecropin reduces LPS-induced lung inflammation.

## 4. Discussion

Overuse of antibiotics has been attributed as a primary factor underlying the emergence and persistence of multidrug-resistant bacteria, promoting their rapid evolution. Particularly, Gram-negative bacteria represent a serious clinical as well as global public health problem, with infections with colistin-resistant Gram-negative bacteria at the top of the World Health Organization priority list as targets for the development of new types of antibiotics [58]. Toward this end, the aim of the present study was to ultimately resolve the problems associated with infection by colistin-resistant bacteria by proposing a new peptide antibiotic. Specifically, we evaluated the potency of *T. ni* cecropin against Gram-negative bacteria, focusing on colistin-resistant bacteria. The results showed that compared with colistin, *T. ni* cecropin exhibited superior activity against colistin-resistant bacteria owing to strong binding interactions with LPS, confirming its therapeutic potential as a peptide antibiotic. To our knowledge, this represents the first report investigating the efficacy of any cecropin against colistin-resistant bacteria and the underlying antimicrobial mechanism of action.

Various attempts have been made to develop peptide antibiotics derived from natural cecropin peptides. Peptides with a short length are more cost effective for therapeutic applications. In particular, 12-meric Pap12-6 peptides designed from the N-terminal 12 amino acids of the insect cecropin papiliocin have demonstrated anti-sepsis activity against *E. coli*-infected mice [55]. Another strategy is to hybridize known peptides with cecropins to improve antibacterial activity while maintaining low cytotoxicity. In this regard, W-BP100 and CAME, conjugated peptides of cecropin and melittin, have the characteristics of active peptides with improved bactericidal power in vitro and in a sepsis animal model [59]. A cecropin A–melittin hybrid peptide has been shown to exhibit antimicrobial activity against multidrug-resistant bacteria in insects in vivo [60]. A hybrid of cecropin A and magainin 2 (CAMA) has demonstrated anti-septic activity with low toxicity [61] and antifungal activity [62]. Finally, PapMA-3, derived from papiliocin and magainin 2, showed outstanding synergistic effects with antibiotics [63].

In this study, *T. ni* cecropin showed promising potency compared to that of cecropin A, polymyxin B, colistin, and melittin against colistin-resistant bacteria, exhibiting the highest relative selectivity index value among all tested peptides. Moreover, *T. ni* cecropin demonstrated superior bactericidal activity against four colistin-resistant bacteria and also inhibited the biofilm formation of ColREC more potently than colistin. Biofilms provide an environment for bacteria to survive and even become adaptable to the medical environment, which is one of the reasons bacteria become resistant to antibiotics [64]. Therefore, blocking biofilm formation is a therapeutic approach to Gram-negative infections. Overall, our data confirmed the superior selectivity of *T. ni* cecropin over that of colistin against colistin-resistant bacteria, with low cytotoxicity against mammalian cells.

In patients with Gram-negative bacterial infection, the presence of circulating LPS induces the excessive production of inflammatory cytokines, which causes serious septic shock. To support its potential as an anti-endotoxin agent, the interaction of the *T. ni* cecropin peptide with LPS was characterized using LAL, BC displacement assays, STD NMR, and ITC measurements. The results showed that *T. ni* cecropin exhibits comparable LPS binding to that of LL-37, a known LPS-binding peptide. As shown in CD spectra, the formation of α-helical structures in membrane-mimetic environments, which constitute important characteristics for the membrane-disrupting properties of AMP [65], may facilitate *T. ni* cecropin permeabilization of the outer membrane of colistin-resistant bacteria. Moreover, the cationic N-terminal helices as well as the hydrophobic C-terminal helix of *T. ni* cecropin may allow it to permeate the ColREC membrane more efficiently than colistin. FE-SEM findings also suggested the membrane-targeting lethality mechanism as being due to bacterial membrane penetration and the resulting death from intracellular fluid efflux. Three-dimensional structural studies using NMR spectroscopy are currently underway. As *T. ni* cecropin uniquely possesses an additional Phe at the fourth position of its N-terminal helix compared to other cecropins, knowledge of the structure–activity relationships will shed light on understanding the mechanism of action and help to develop short novel peptide antibiotic derivatives based on structure–activity relationships.

We found that *T. ni* cecropin significantly inhibited the release of nitrite and IL-6 in murine macrophage RAW 264.7 cells stimulated with LPS as well as in ColREC-infected murine macrophages. Furthermore, by stimulating macrophages with agonists for various TLRs, we demonstrated that *T. ni* cecropin specifically regulates TLR4 downstream signaling, which is activated by LPS stimulation. Inhibition of TLR4-mediated inflammatory signaling by *T. ni* cecropin was further confirmed by the inhibition of SEAP activity as well as macrophage presentation of TLR4 receptors as determined using flow cytometry. In addition, SPR measurements revealed the micromolar binding affinity of *T. ni* cecropin for the TLR4/MD2 protein complex. Together, these results imply that *T. ni* cecropin not only inhibits cell surface expression of TLR4 as well as the LPS-induced TLR4 signaling cascade by directly binding to LPS but may also inhibit LPS binding to TLR4/MD2 by direct interactions with the TLR4/MD2 complex. Analysis of the downstream TLR4 signaling pathway should be further elucidated to understand the detailed mechanism using immunoblotting or reverse transcription polymerase chain reaction.

The results of evaluating *T. ni* cecropin toxicity against mammalian cells demonstrated the biocompatibility of this peptide, thereby addressing potential safety concerns regarding the future therapeutic application of *T. ni* cecropin in the clinic. Moreover, *T. ni* cecropin neutralized LPS and efficiently suppressed inflammatory cytokine levels in the serum of an endotoxemia mouse model, confirming its therapeutic potential to treat Gram-negative sepsis. Additionally, reduced levels of AST, ALT, and BUN confirmed *T. ni* cecropin’s ability to alleviate liver and kidney damage. Furthermore, *T. ni* cecropin alleviated LPS-induced inflammation in the lungs. These findings are consistent with those of *Aedes aegypti* cecropin, which suppressed the inflammatory response in mice infected with *E. coli* or *P. aeruginosa* and exhibited anti-sepsis activity with organ protection [40]. Papiliocin showed anti-septic effects in an *E. coli* K1-septic mouse model, alleviating the inflammatory response and organ damage [37]. Nevertheless, prior to therapeutic and clinical application, the in vivo therapeutic potency of *T. ni* cecropin needs to be further confirmed by establishing an animal model of infection induced by a colistin-resistant pathogen.

## 5. Conclusions

Our study suggested a basis for the development of peptide antibiotics against colistin-resistant bacteria. *T. ni* cecropin demonstrated good antibacterial and antibiofilm activity against Gram-negative bacteria, showing that it fulfills the requirements of a peptide antibiotic. It exhibited strong membrane permeability and potent antibacterial activity even against colistin-resistant bacteria by targeting the outer membrane of Gram-negative bacteria via strong binding to LPS. In addition, *T. ni* cecropin exerts anti-inflammatory effects, inhibiting inflammatory responses to LPS and ColREC stimulation. This effect was found to be based on the inhibition of TLR4-mediated inflammatory signaling. Notably, *T. ni* cecropin also exhibited LPS-neutralizing and immunosuppressive activities in an endotoxemia mouse model, implying that *T. ni* cecropin has considerable potential as a potent anti-septic peptide. Taken together, our results support the idea that *T. ni* cecropin may be a promising starting point for the development of novel peptide antibiotic alternatives to polymyxins for the treatment of colistin-resistant Gram-negative infections.

## Figures and Tables

**Table 1:** Minimum inhibitory concentration (MIC) of antimicrobial peptides against various microorganism.

	Minimal Inhibitory Concentration (μM)				
Microorganism	Cecropin A	*T. ni* Cecropin	Polymyxin B	Colistin	Melittin
*E. coli*	2	2	0.25	0.25	8
*A. baumannii*	2	1	0.5	0.25	4
*P. aeruginosa*	8	4	2	1	32
*K. pneumoniae*	1	1	0.25	0.25	32
ColREC 1557	1	1	8	8	2
ColREC 12	1	1	4	4	2
ColRAB 1915	1	1	64	>64	2
ColRKP 139	2	2	64	>64	32
GM *	2.25	1.63	17.88	33.72	14.25
HC_10_^†^	200	200	200	200	3.1
Relative selective Index **	88.89	123.08	11.19	5.93	0.22

* Geometric mean (GM) represents the average MIC value of all bacterial strains. ^†^ Hemolysis concentration (HC)_10_ is the concentration that causes 10% hemolysis in sheep red blood cells. ** Relative selective index was calculated as HC_10_/GM. For the calculation, 200 μM was applied to the formula if no hemolysis was observed at 100 μM. For MIC values above 64 μM, 128 μM was used in the calculations. Higher relative selective index values indicate higher cell selectivity. ColREC: colistin-resistant *Escherichia coli (E. coli)*; ColRAB: colistin-resistant *Acinetobacter baumannii (A. baumannii)*; ColRKP: colistin-resistant *Klebsiella pneumoniae (K. pneumonia)*.

## Data Availability

The data presented in the manuscript are available on request from the corresponding author.

## Supplementary Materials

The following supporting information can be downloaded at: https://www.mdpi.com/article/10.3390/pharmaceutics15061752/s1, Figure S1. Characterization of synthesized *T. ni* cecropin by high-performance liquid chromatography (HPLC) and mass spectrometry (MS).
