# Supplementary material for: Characterization of the Antimicrobial Activities of Trichoplusia ni Cecropin A as a High-Potency Therapeutic against Colistin-Resistant Escherichia coli
Source: Pharmaceutics. 2023 Jun 16;15(6):1752. doi: 10.3390/pharmaceutics15061752 (PMC10304470; doi:10.3390/pharmaceutics15061752)
Supplement: Supplementary file 1 [file pharmaceutics-15-01752-s001.zip › pharmaceutics-2447337-supplementary.pdf]

pharmaceutics

Supplementary Materials: Characterization of the Antimicrobial Activities of *Trichoplusia ni* Cecropin A as a High-potency Therapeutic against Colistin-resistant *Escherichia coli*

Hyeju Lee, Byeongkwon Kim, Minju Kim, Seoyeong Yoo, Jinkyeong Lee, Eunha Hwang and Yangmee Kim

This PDF file includes:

Figure S1. Characterization of synthesized *T. ni* cecropin by high-performance liquid chromatography (HPLC) and mass spectrometry (MS). (**a**) HPLC chromatogram of synthesized *T. ni* cecropin. (**b**) MS chromatogram of synthesized *T. ni* cecropin. m/z indicates mass divided by charge number.

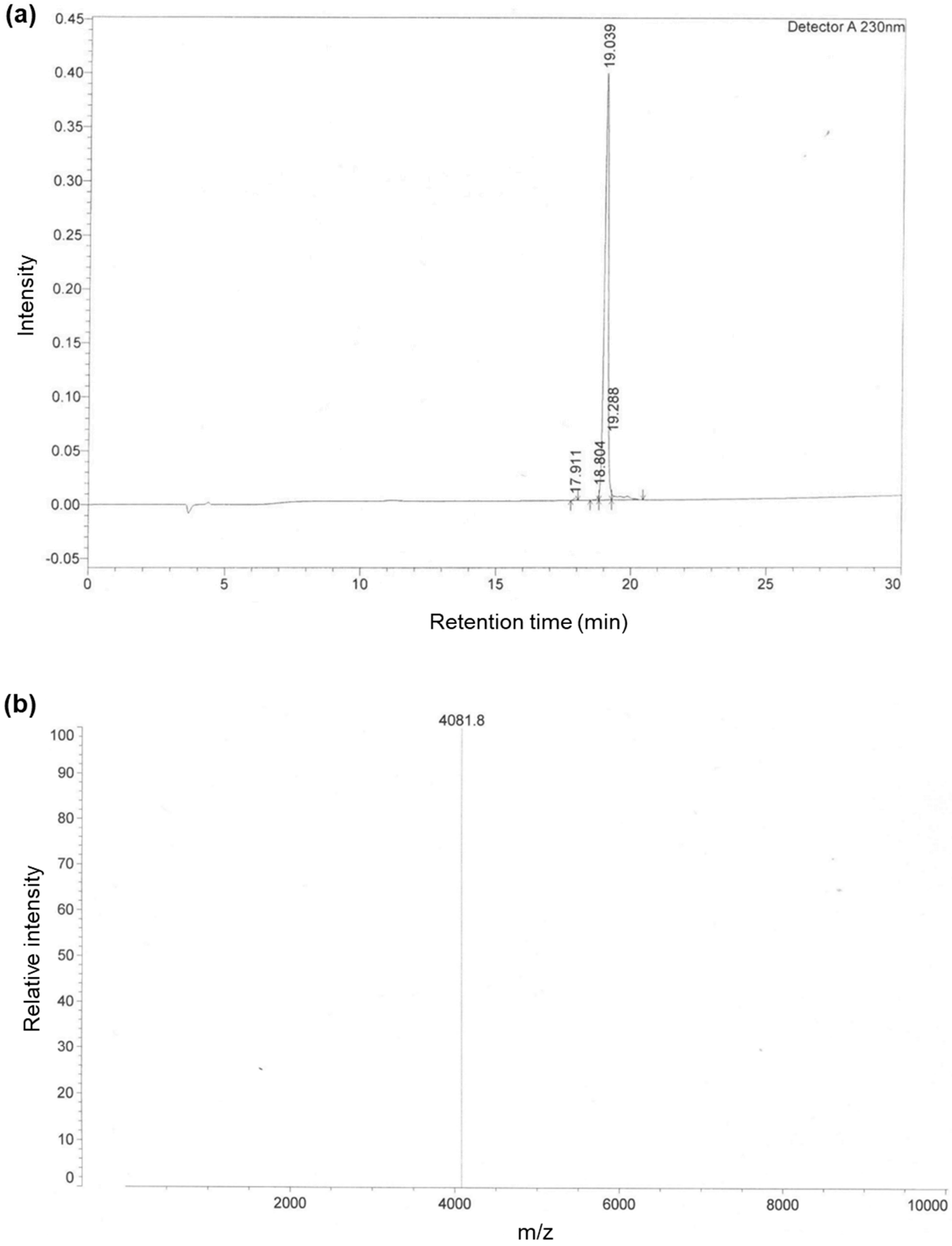

Figure S1. Characterization of synthesized *T. ni* cecropin by high-performance liquid chromatography (HPLC) and mass spectrometry (MS). (**a**) HPLC chromatogram of synthesized *T. ni* cecropin. (**b**) MS chromatogram of synthesized *T. ni* cecropin. m/z indicates mass divided by charge number.
